# Asymptomatic norovirus infection associated with swimming at a tropical beach: A prospective cohort study

**DOI:** 10.1371/journal.pone.0195056

**Published:** 2018-03-28

**Authors:** Timothy J. Wade, Swinburne A. J. Augustine, Shannon M. Griffin, Elizabeth A. Sams, Kevin H. Oshima, Andrey I. Egorov, Kaneatra J. Simmons, Tarsha N. Eason, Alfred P. Dufour

**Affiliations:** 1 Office of Research and Development, United States Environmental Protection Agency, Research Triangle Park, NC, United States of America; 2 Office of Research and Development, United States Environmental Protection Agency, Cincinnati, OH, United States of America; 3 Oconee Fall Line Technical College, Dublin, GA, United States of America; Australian National University, AUSTRALIA

## Abstract

**Background:**

Swimming in fecally-contaminated waterbodies can result in gastrointestinal infections. However, the pathogenic microorganisms responsible are not well understood because sporadic cases of illness are not reported completely, exposure information is often not collected, and epidemiology studies rely on self-reported symptoms. Noroviruses are considered a likely cause because they are found in high densities in sewage, resistant to wastewater treatment and survive in the environment. In this study, saliva samples were collected from subjects at a beach in Puerto Rico and tested for evidence of norovirus-specific IgG responses as an indicator of incident norovirus infection.

**Methods:**

Saliva samples were collected from 1298 participants using an oral swab. Samples were collected on the day of the beach visit (S1); after 10–12 days (S2); and after three weeks (S3). Saliva was tested for IgG responses to GI.1 and GII.4 noroviruses using a microsphere based multiplex salivary immunoassay. Immunoconversion was defined as a four-fold increase in median fluorescence intensity (MFI) from S1 to S2 with the S3 sample at least three times above the S1 MFI.

**Results:**

Thirty-four subjects (2.6%) immunoconverted to GI.1 or GII.4 norovirus. Swimmers who immersed their head in water had a higher rate of immunoconversion (3.4%), compared to either non-swimmers (0.0%, p = 0.003) or waders and non-swimmers combined (0.4%, Odds Ratio: 5.07, 95% Confidence Interval:1.48–17.00). Immunoconversion was not associated with gastrointestinal symptoms.

**Conclusions:**

This is the first study to demonstrate an association between swimming at a beach impacted by fecal contamination and asymptomatic norovirus infection. The findings implicate recreational water as potentially important transmission pathway for norovirus infection.

## Introduction

Swimming in fecally-contaminated natural waters can result in gastrointestinal infections caused by a range of pathogenic microorganisms [1]. Since the 1950s, epidemiology studies have observed excess gastrointestinal symptoms among swimmers in fecally-contaminated waterbodies under non-outbreak conditions, but have not identified causative waterborne pathogens [2]. For over 20 years, researchers hypothesized that viruses, in particular norovirus (NoV), may be a major cause of swimming-associated gastroenteritis [3] due to their resistance to waste water treatment[4], ability to survive in the environment [5], low infective dose [5], high concentrations in sewage [4] and the short time lag between swimming exposure and symptoms, characteristic of viral gastroenteritis [6].

NoV infection is one of the most common causes of gastroenteritis, responsible for an estimated 699 million illnesses annually [7]. Most adults have detectable antibodies to NoV [8]. NoV are single-stranded RNA viruses, classified into seven genogroups (GI-GVII), with GI and GII responsible for most human infections [5]. NoVs are transmitted via the fecal-oral route by person to person transmission and contaminated food or water. Outbreaks have been associated with a variety of settings including child care centers, nursing homes, hospitals, cruise ships and restaurants [5]. Illnesses are characterized by sudden onset of vomiting and/or diarrhea within 12–48 hours of exposure lasting 2–3 days [5]. Immunity is short term, though repeated exposures may generate long-term resistance [5]. NoV infections typically peak in the winter but transmission occurs year-round, including summertime outbreaks associated with lakes [9].

Salivary immunoglobulins (Ig) specific to NoV can be effective in detecting recent NoV infections [10, 11]. Following an inoculation challenge with Norwalk (GI.1) virus four-fold increases in specific salivary IgG and IgA responses were evident eight and four days post-challenge, respectively [11]. Previously, we used saliva samples from a subset of participants of a volunteer challenge study [12] to develop a multiplex immunoassay and demonstrated 100% accuracy in discriminating GI.1 infected and non-infected volunteers [10].

In 2009, we conducted an epidemiology study at a beach in Puerto Rico. As part of this study, saliva samples were collected from a subsample of participants [13]. The objective of the present study was to use salivary antibody responses as biomarker of infection to evaluate the risk of NoV transmission and swimming exposure.

## Methods

### Epidemiology study

In 2009, we conducted a prospective study of the acute health risks associated with swimming at a tropical beach. The study design has been previously described [13]. Briefly, we enrolled beachgoers attending Boquerón Beach on the western coast of Puerto Rico on summer weekends. Unaccompanied minors or those who could not speak English or Spanish were ineligible. Upon leaving the beach, subjects were interviewed about water contact and other activities during the visit to the beach. Ten to 12 days later, a participating adult was interviewed by telephone about symptoms experienced by household members. We collected serial saliva samples from a subgroup of the 15,726 subjects who completed the interview portion of the study. Approximately 100 households were offered enrollment in the saliva study daily, with a goal of enrolling a total of 2500 subjects. Enrollment in the saliva study was offered to all households participating in the interview portion of the study each day of the study until the approximate daily quota of 100 households was reached.

The study was approved by the Institutional Review Board for the University of North Carolina at Chapel Hill. Approval to conduct the study was also obtained from local and regional authorities including: US EPA Caribbean Division, Puerto Rico Department of Natural and Environmental Resources, Cabo Rojo Mayor’s Office and Boquerón Beach Park. Among those participating in the saliva collection protocol, adults 18 years and older provided signed consent for themselves and for their children under 7. Children aged 7–17 provided signed assent along with signed parental permission. Infants under one year were excluded from saliva collection due to the presence of maternal antibodies, high rates of non-waterborne infection and lack of crevicular fluid (the exudate between the teeth and gums enriched with serum components, including serum IgG).

### Water sample collection and testing

At 8:00 AM, 11:00 AM and 3:00 PM each study day, six water samples were collected at representative locations as described previously [13, 14]. Samples were tested for fecal contamination by measuring culturable enterococci as described previously [13, 14], using US EPA Method 1600 [15].

### Saliva collection

Samples were collected using an Oracol^TM^ sampler (Malvern Medical Developments, United Kingdom), which consists of a cylindrical sponge with a handle. Collection involves gentle rubbing of the gums to stimulate saliva and crevicular fluid production (hereafter called “saliva samples”) for approximately one minute or until the sponge is saturated.

Subjects provided three saliva samples: A baseline (S1) sample upon leaving the beach, a second sample (S2) at 10–14 days and a third sample (S3) 30–40 days following the beach visit. Upon leaving the beach, subjects were given sampling kits, shipping containers with ice packs and return shipping boxes. Following collection, subjects placed samples in a cooler and arranged for shipment to EPA. Upon delivery, samples were centrifuged to extract saliva from the sponge along with debris from the saliva, and stored at -80^o^ C until analysis.

### Analysis of saliva samples

Two unique sets of Luminex microspheres were coupled to NoV antigens as described previously [16]. We used recombinant antigens (P domain of the major capsid protein) from NoV GI.1 (Norwalk virus) and GII.4 (VA387 variant) NoV obtained from Xi Jiang of Cincinnati Children’s Hospital Medical Center. The GII.4 strain VA387 represents the most common NoV genotype, and the GI.1 Norwalk strain causes a substantial proportion of waterborne NoV outbreaks [5, 10]. Antigens were produced using an *E*. *coli* expression system described previously [17]. A third set of microspheres, which was not coupled to a viral antigen, was blocked with bovine serum albumin (BSA) as an internal control.

Three sets of NoV protein-coupled and control microspheres were added to wells of MultiScreen BV 1.2 μm filter microplates (Millipore, Inc., Billerica, MA) and incubated with saliva diluted 1:8 in PBS-1% BSA for 30 minutes. Plates were processed as described previously [18] using 8 μg/mL of biotin-labeled affinity purified goat anti-human IgG detection antibody (KPL, Gaithersburg, MD) and 12 μg/mL streptavidin-phycoerythrin conjugate (SAPE; Invitrogen, Carlsbad, CA). Microplates were analyzed using a Luminex LX100 at default settings; net (background subtracted), median fluorescence intensity (MFI) of the reporter was estimated from at least 100 microspheres of each type and used in data analysis. Background responses were obtained from NoV protein-coupled microspheres incubated with buffer in place of saliva.

### Immunoconversion definition

The outcome was an immunoconversion to GI.I or GII.4 NoV between the beach visit and S2 saliva collection 10–14 days later. Immunoconversion was defined as a four-fold increase in net anti-NoV IgG antibody (measured in MFI) in S2 compared to S1. Because anti-NoV IgG remains elevated one month or more after infection [10, 11], S3 MFI was required to be at least three-fold above the S1 MFI. The S2 response was required to be above a minimum of MFI of 505 and an age-specific minimum cutoff value described below. The minimum 505 MFI was determined as the antilog of the mean MFI of the control microspheres plus 3 standard deviations of the log_10_ transformed data (10^mean(h)+3SD(h)^), where h = log_10_(control MFI), as described previously [18].

To reduce potential false-positives resulting from variability in salivary IgG and the tendency for anti-NoV IgG to increase with age we also incorporated an age-specific MFI minimum for S2 samples. We modeled log_10_ transformed anti-NoV-MFI IgG response as a restricted cubic spline function of age with five knots as recommended by Harrell [19] and estimated the upper bound of the 75% prediction interval. To be considered an immunoconversion, the S2 MFI value had to exceed both 505 and the age-specific upper 75% prediction interval.

### Exposure

The exposure of interest was swimming, defined as head immersion in water, consistent with how we defined swimming in previous studies [14, 20]. Three exposure categories were generated: 1) Head immersion swimmers (“Swimmers”), were those who reported submerging their head in water; 2) “Non-swimmers” were those who reported no water contact; and 3) “Waders” were those who swam but did not immerse their head. Fecal contamination in water measured by the geometric mean of culturable enterococci (colony forming units/ml, or CFU/ml) was also analyzed as a predictor of immunoconversion. We also considered alternate definitions of swimming including those who had any contact with water and those who immersed a minimum of their body (not necessarily their head) in water.

### Data analysis

Immunoconversion status was cross-tabulated with demographic factors and suspected risk factors for NoV infection including: household size, consumption of undercooked meat or raw fish, contact with other ill people, animal contact, digging in or being buried in the sand, contact with algae, water temperature, rainfall within 18 hours before the beach visit, other swimming prior to or after the beach visit and presence of other chronic health conditions. Two-sided p-values were calculated using Fisher’s Exact test.

Factors associated with immunoconversion in tabulations or those suspected to be associated with swimming exposure and NoV infection were included in a multivariable logistic regression model where the outcome was NoV immunoconversion. To examine the association between water quality and NoV immunoconversion, the daily log_10_ enterococci geometric mean was modeled as a continuous measure and analyses were restricted to head immersion swimmers. Final models were selected by sequential backwards selection, minimizing Akaike’s Information Criterion [21]. Goodness of fit was confirmed using the Hosmer-Lemeshow goodness of fit test [22]. Data analysis was conducted in Stata version 14.2 (Stata Corporation, College Station TX).

## Results

### Study participants

The vast majority of the study population were Puerto Rico residents (98%). A total of 2372 subjects from 1209 households were enrolled in the saliva collection protocol. These subjects returned 5533 samples. After excluding samples with illegible or invalid identification numbers, 5455 saliva samples from 2237 subjects remained. Of these subjects, 1407 returned all three samples. Following analysis, 31 samples from 23 subjects were excluded due to high background levels (>500 MFI on control beads), possibly due to gum disease or irritation causing blood in the sample. An additional 86 subjects were excluded because they did not complete all questionnaires, leaving 1298 subjects with three samples and questionnaire data available for analysis. The median time between the S2 and S1 sample collection and S3 and S1 collection was 11 and 31 days, respectively.

### Immunoconversions to noroviruses

**Fig 1:**
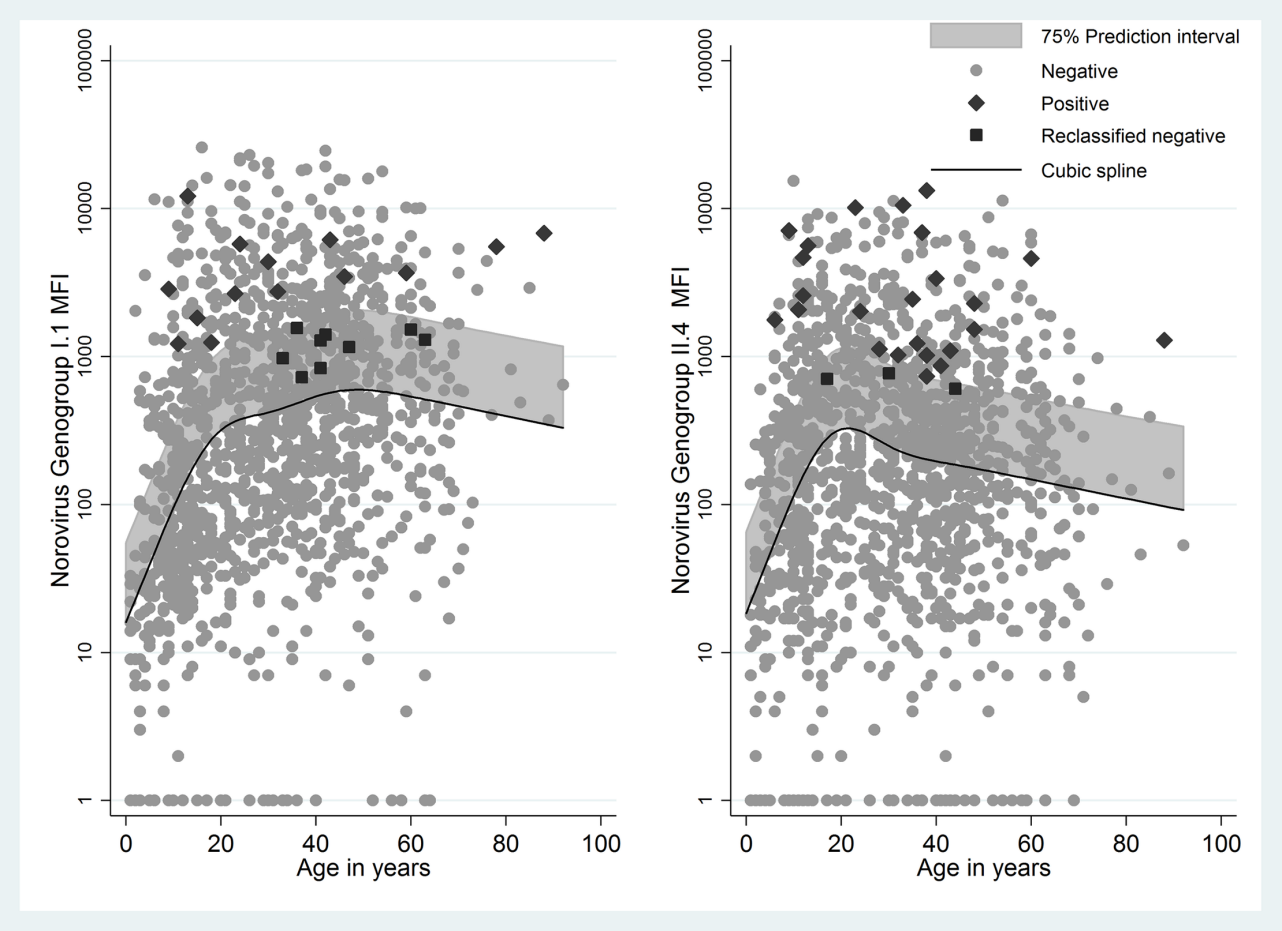
Anti-Norovirus salivary IgG response at S2 measured as median fluorescence intensity. (a) Anti-Norovirus GI.1 salivary IgG response at S2, cubic spline (5 knot) function of age, and age-specific upper 75% prediction interval. The term “Positive” is used to denote individuals who immunoconverted and the term “Negative” for individuals who did not immunoconvert. (b) Anti-Norovirus GII.4 salivary IgG response at S2, cubic spline (5 knot) function of age, and age-specific upper 75% prediction interval. The term “Positive” is used to denote individuals who immunoconverted and the term “Negative” for individuals who did not immunoconvert.

Anti-NoV GI.1 and GII.4 responses in S2 samples as non-linear functions of age and 75% prediction intervals are shown in Fig 1. Anti-NoV IgG responses increased until approximately 20 years of age, and then leveled off. The age-specific minimum response criterion resulted in nine reclassified as negative for GI.1 NoV and three reclassified as negative for GII.4 NoV and (Fig 1).

**Fig 2:**
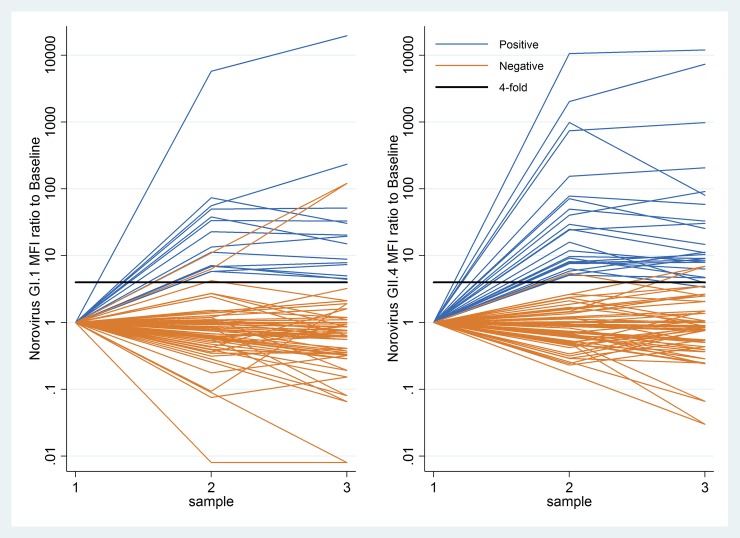
MFI ratios of salivary IgG responses to NoV at S2 (10–14 days) and S3 (30–40 days) to baseline (S1). **(a)** GI.1 NoV IgG ratios to S1 for those who met immunoconversion criteria (blue lines) and a random sample (N = 30) of those who did not (orange lines). Solid horizontal line is shown at minimum S2/S1 ratio of four. The term “Positive” is used to denote individuals who immunoconverted and the term “Negative” for individuals who did not immunoconvert. **(b)** GII.4 NoV IgG ratios to S1 for those who met immunoconversion criteria (blue lines) and a random sample (N = 30) of those who did not (orange lines). Solid horizontal line is shown at minimum S2/S1 ratio of four. The term “Positive” is used to denote individuals who immunoconverted and the term “Negative” for individuals who did not immunoconvert.

Thirty-four subjects (2.6%) immunoconverted to GI.1 or GII.4 NoV including 14 to GI.1 (1.1%), 24 to GII.4 (1.9%); and four to both (Table 1). Fig 2 illustrates ratios of anti-NoV GI.1 and GII.4 IgG responses to baseline (S1) for those who immunoconverted and a random sample (N = 30) of those who did not, including several responses which met the four-fold criterion but were classified as negative because they did not meet either the minimum MFI or S3 to S1 ratio criteria.

**Table 1:** Factors associated with NoV immunoconversions^a^.

	GI.1 N(%)	GII.4 N(%)	GI.1 or GII.4 N(%)
**All** (N = 1298)	14 (1.1)	24 (1.9)	34^d^ (2.6)
**Age**			
0–4 (N = 48)	0 (0.0)	0 (0.0)	0 (0.0)
5–11 (N = 148)	2 (1.4)	3 (2.0)	5 (3.4)
12–19 (N = 210)	3 (1.4)	3 (1.4)	6 (2.9)
20–34 (N = 319)	4 (1.3)	5 (1.6)	6 (1.9)
35-over (N = 568)	5 (0.9)	13 (2.3)	17 (3.0)
p-value	0.90	0.89	0.69
**Sex**			
Male (N = 547)	6 (1.1)	12 (2.4)	16 (2.9)
Female (N = 751)	8 (1.1)	12 (1.9)	18 (2.4)
p-value	1	0.53	0.60
**Consumed raw or undercooked meat**			
No (N = 1241)	12 (0.97)	22 (1.8)	31 (2.5)
Yes (N = 57)	2 (3.5)	2 (3.5)	3 (5.3)
p-value	0.12	0.28	0.18
**Other swimming after beach visit**^**b**^			
No (N = 785)	6 (0.8)	10 (1.3)	13 (1.7)
Yes (N = 482)	7 (1.5)	13 (2.7)	20 (4.2)
p-value	0.26	0.08	0.01
**Rainfall (inches) during previous 18 -hours**			
0–0.5 (N = 1245)	12 (1.0)	20 (1.6)	28 (2.3)
> = 0.5 (N = 53)	2 (3.8)	4 (7.5)	6 (11.3)
p-value	0.11	0.01	0.0019
**Chronic GI illness**			
No (N = 1224)	10 (0.8)	21 (1.7)	28 (2.3)
Yes (N = 74)	4 (5.4)	3 (4.1)	6 (8.1)
p-value	0.006	0.15	0.01
**Diarrhea**^**c**^			
No (N = 1248)	13 (1.0)	23 (1.8)	33 (2.6)
Yes (N = 20)	0 (0.0)	0 (0.0)	0 (0.0)
p-value	1.0	1.0	1.0
**Vomiting**^**c**^			
No (N = 1263)	13 (1.0)	23 (1.8)	33 (2.6)
Yes (N = 5)	0 (0.0)	0 (0.0)	0 (0.0)
p-value	1	1	1
**Fever**^**c**^			
No (N = 1231)	13 (1.1)	23 (1.9)	33 (2.7)
Yes (N = 37)	0 (0.0)	0 (0.0)	0 (0.0)
p-value	1	1	1.0
**Stomachache**^**c**^			
No (N = 1219)	13 (1.1)	22 (1.8)	32 (2.6)
Yes (N = 49)	0 (0.0)	1 (2.0)	1 (2.0)
	1	0.6	1.0

^a^ Numbers for all categories do not add 1298 due to individual non-response. One S2 sample was not tested for G1.1 NoV

^b^ Reported head-immersion swimming after the day of enrollment at the beach^c^ Reported symptom at the 10–12 day follow up interview

^d^ Four individuals immunoconverted to both GI.1 and GII.4

Children 5–11 years of age had the highest percentage of immunoconversions (3.4%, Table 1). Rainfall in the prior 18 hours, swimming after the beach visit and a chronic gastrointestinal condition were associated with NoV immunoconversion. Gender, diarrhea or vomiting within 48 hours prior to the beach visit, other ill household members, consumption of undercooked or raw meat, consumption of shellfish, digging in sand, swimming in the prior week, contact with animals, and a child under five years of age in the household were not associated with NoV immunoconversion.

Twenty subjects (1.6%) reported diarrhea and 5 (0.4%) reported vomiting in the 10–12 days after the beach visit. Of those who immunoconverted, only one reported a symptom (stomachache) during the 10–12 day follow up. There were no significant differences in the frequency of symptoms among those who did and did not immunoconvert (Table 1).

### Swimming and water-associated exposures

Most subjects (84%) reported contact with water and contact was often for extended periods (median 120 minutes). Among those reporting water contact 82% (903/1098) immersed their head in water. Among the 199 non-swimmers there were no immunoconversions to either GI.1 or GII.4 NoV. Three waders immunoconverted to either GI.1 or GII.4 NoV (1.5%) but this did not differ significantly from non-swimmers (p = 0.12). Swimmers who immersed their head had a higher frequency of NoV (GI.1 or GII.4) immunoconversion (3.4%), compared to non-swimmers (p = 0.003, Table 2) and to non-swimmers and waders combined (3.4% vs. 0.8%, p = 0.004, Table 2).

**Table 2:** NoV immunoconversions and swimming exposures.

	GI.1 N(%)	GII.4 N(%)	GI or GII.4 N(%)
**Non-swimmers (N = 199)**	0 (0.0)	0 (0.0)	0 (0.0)
**Waders** (Water contact without head immersion, N = 195)	1 (0.5)	2 (1.0)	3 (1.5)
**Swimmers** (Water contact with head immersion, N = 903)	13 (1.4)	22 (2.4)	31 (3.4)
**p-value**^**a**^	0.14	0.02	0.003
**p-value**^**b**^	0.08	0.02	0.004

^a^ Swimmers vs. non-swimmers (Fisher’s exact test)

^b^ Swimmers vs. non-swimmers and waders combined (Fisher’s exact test)

Because there were no immunoconversions among non-swimmers, we compared head immersion swimming to non-swimmers and waders combined to allow adjustment for covariates by logistic regression. The association between head immersion swimming and NoV immunoconversion strengthened after adjustment (Table 3). In the adjusted model, the odds ratio (OR) and 95% confidence interval (CI) for head immersion swimming and NoV immunoconversion was 5.07 (95% CI: 1.48–17.00) compared to 4.63 (95% CI: 1.41–15.25) without adjustment. Because of missing data in covariates, the adjusted estimate is based on 36 fewer observations than the unadjusted estimate. Restricting the unadjusted estimate to the 1,261 observations also included in the adjusted model, resulted in a slightly lower OR of 4.55 (95% CI: 1.38–15.00). Rainfall in the prior 18 hours, head-immersion swimming following the beach visit, and a chronic gastrointestinal condition were also risk factors for NoV immunoconversion (Table 3). Although the association was not statistically significant, NoV immunoconversion among those who immersed their head was positively associated with fecal contamination as measured by enterococci (OR = 2.43 per 10-fold increase in enterococci; 95% CI = 0.69–8.58, see S1 Fig).

**Table 3:** Risk factors for NoV immunoconversion- logistic regression models.

Immunoconversion to either GI.1 or GII.4 NoV	Unadjusted model^a^ Odds ratio (95% Confidence interval) p-value	Adjusted model^b^ Odds ratio (95% Confidence interval) p-value
**Head Immersion**	4.63 (1.41–15.25) 0.012	5.07 (1.50–17.12) 0.009
**Age (year)**		1.02 (1.00–1.04) 0.11
**Chronic GI**		4.21 (1.63–10.88) 0.003
**Rain (per ¼ inch)**		1.50 (1.07–2.11) 0.02
**Other swimming**		2.48 (1.20–5.13) 0.014
**Head Immersion (GI.1 only)**	5.75 (0.75–44.08) 0.09	6.70 (0.83–53.65)^c^ 0.07
**Head Immersion (GII.4 only)**	4.89 (1.15–20.92) 0.03	5.34 (1.21–23.51)^c^ 0.03

^a^ N = 1,297 (one missing observation for head immersion swimming)

^b^ N = 1,261 (sample size is reduced further due to missing data on covariates)

^c^Adjusted for chronic GI condition, rainfall, other swimming, and age

Those who any contact with water (head immersion swimmers and waders combined) and those who at a minimum immersed their body in water (not necessarily their head) also had a higher frequency of NoV immunoconversion (3.1%, S1 Table) relative to non-swimmers (p = 0.006). However, it is difficult to distinguish among these different exposure categories because the majority of swimmers immersed their head and all but three of NoV immunoconversions were among swimmers who immersed their head.

## Discussion

We found approximately five times higher rates of immunoconversion to two common NoVs among those immersing their head in the water at a tropical marine beach compared to non-swimmers and waders who did not. These findings provide evidence that during non-outbreak conditions, NoV infections can be transmitted through swimming, even in the absence of symptoms.

The beach was located near a municipal sewage discharge and several smaller discharges which may have been sources of fecal contamination. NoVs are present in high densities in treated and untreated sewage [4], are resistant to environmental degradation in water [23] and have a low infectious dose [5]. In addition, several water samples from the beach, nearby lagoons, and wastewater effluent identified NoV (genogroups I and II) by PCR during the time of the study [24]. Although we observed a trend between immunoconversion and levels of fecal contamination in the water (S1 Fig) we cannot be certain whether the source of infection was environmental (e.g., sewage discharges or other runoff), or shedding of virus from swimmers, particularly from diaper-age children. At least one child under three years of age was represented in our study population each day, and nearly all (97%) entered the water. This beach had a high density of swimmers who remained in the water for extended periods and had little wave action, potentially increasing the risk for NoV transmission. Even asymptomatic infected individuals can have high densities of NoV in stool (10^5^−10^9^ per gram of feces) [25] and swimmers without symptoms can shed approximately 0.14 grams of fecal material per swimming event [26].

Nearly all immunoconversions were unaccompanied by gastrointestinal symptoms. While this was unexpected, there is evidence for a high prevalence of asymptomatic NoV infection. In some studies, particularly in lower income areas, healthy controls had a higher frequency of NoV detection in their stool than symptomatic cases [27]. Acquired immunity from repeated exposures may be protective of symptoms but not infection [27] and it is possible that this population, which was almost entirely made up of Puerto Rico residents (98%), may have had repeated exposures to NoV since an early age. Also, a few mouthfuls of water may have resulted in a low inoculum, which can cause subclinical infections that produce no or mild symptoms [27]. It is also possible that the interview did not fully capture gastrointestinal symptoms which were notably less frequent compared to similar studies [14].

There is innate resistance to most NoVs among the 20–30% of the population that does not secrete ABO histo-blood group antigens (non-secretors), as these are important receptors for NoV. Non-secretors do not develop antibody responses so could not account for asymptomatic infections we observed [28]. We were unable to assess secretor status in our study due to the limited volume of saliva. However, we would not expect secretor status to differ with regard to swimming exposures. Therefore, the inclusion of non-secretors likely diluted the overall effect estimate we observed (biased it towards the null). While the results of this analysis are applicable to the general population consisting of secretors and non-secretors, the effects of swimming among secretors only could be slightly stronger. We are unaware of studies that have documented the prevalence of non-secretors in Puerto Rico, but one study suggested non-secretors may be less prevalent in those of Mesoamerican ancestry compared to European or African ancestry [28]. Another study in an Ecuadorian cohort found only 12% to be non-secretors [29].

Other risk factors implicated waterborne NoV transmission. Additional head-immersion swimming following the beach visit and rainfall the day prior to the visit were both associated with immunoconversion. Rainfall can adversely impact water quality by increasing runoff [30] and bypasses of sewage treatment, and has been linked to waterborne disease outbreaks [31].

Preferably, infection would be confirmed in stool, but the collection and shipment of stool is challenging. Saliva samples are non-invasive, can be self-collected and returned via express mail without preservation making them ideal to study infections in large cohorts. Saliva can exhibit variability in non-specific binding which could result in false-positive responses. To increase outcome specificity, we used a stringent definition of immunoconversion. Relaxing the definition by dropping the age-specific cutoff resulted in 11 more immunoconversions and slightly attenuated the association with head immersion swimming (adjusted OR: 4.25; 95% CI: 1.62–11.17). Although we cannot rule out that some immunoconversions were false-positives due to imperfect specificity, misclassification of immmunoconversion status would be random with regard to swimming, and as a result, bias our observed effect estimates toward a null association. With random misclassification, we would expect the true association between swimming and NoV infection to be even stronger than what we observed.

There are several other limitations that should be considered. The salivary immunoassay used two recombinant NoV antigens representing strains which may not have been prevalent at the time of the study. Although previous research has demonstrated cross-reactivity in antibody responses to heterologous NoVs within the same genogroup [32], such responses may be weaker resulting in reduced sensitivity. The study relied on self-reporting of swimming and gastrointestinal symptoms which may have resulted in misclassification, but we also expect these to be random with regard to NoV immunoconversion. Because this was an observational study and there few immunoconversions in the unexposed groups, it is possible that we were unable to fully control for other risk factors for NoV infection. However, it is unlikely such bias could fully explain the associations we observed. We were able to account for many known risk factors for NoV infection including likely foodborne exposures such as consumption of raw or undercooked meat or fish, contacts with other ill people, young children in the household, and contact with animals. These factors were not strongly associated with either swimming exposure or norovirus infection in our data, and thus were not likely to bias our results. Moreover, the adjusted and unadjusted ORs for the association between head immersion swimming and NoV immunoconversion did not differ substantially (4.63 and 5.07 for the unadjusted and adjusted ORs, respectively).

Waterborne NoV outbreaks are well documented [9] but endemic waterborne transmission of NoV is less well described. These results provide evidence that recreational waterborne transmission may play an important role in NoV disease dynamics and may contribute to sustaining a high level of infection prevalence. These findings may have implications for improved wastewater treatment which is more effective in treating viral waterborne pathogens and the further development of water quality indicators that better mimic the fate and transport of viruses in the environment. Future studies may consider focusing on children who may be particularly susceptible to swimming- associated illnesses and infection. Further efforts should also consider the continued development of a multiplexed salivary immunoassay for NoV incorporating that can capture a wide range of common NoV variants.

This study took place in Puerto Rico nearly ten years ago in 2009 under considerably different conditions than the island faces today. Due to the damage to drinking water and wastewater infrastructure from Hurricane Maria in 2017, it is likely that beaches, streams, rivers and drinking water have been further impacted by untreated or inadequately treated sewage. Although we have no data to directly support this, these impacts could also potentially increase the transmission rate of NoV, as well as other waterborne infections, in the population.

## Supporting information

S1 Fig Probability of norovirus immunoconversion among head immersion swimmers. (PDF)

S1 Table Swimming exposure and NoV immunoconversion for alternate swimming definitions. (DOCX)
