# Supplementary material for: Asymptomatic norovirus infection associated with swimming at a tropical beach: A prospective cohort study
Source: PLoS One. 2018 Mar 28;13(3):e0195056. doi: 10.1371/journal.pone.0195056 (PMC5874074; doi:10.1371/journal.pone.0195056)
Supplement: S1 Fig — (PDF) [file pone.0195056.s001.pdf]

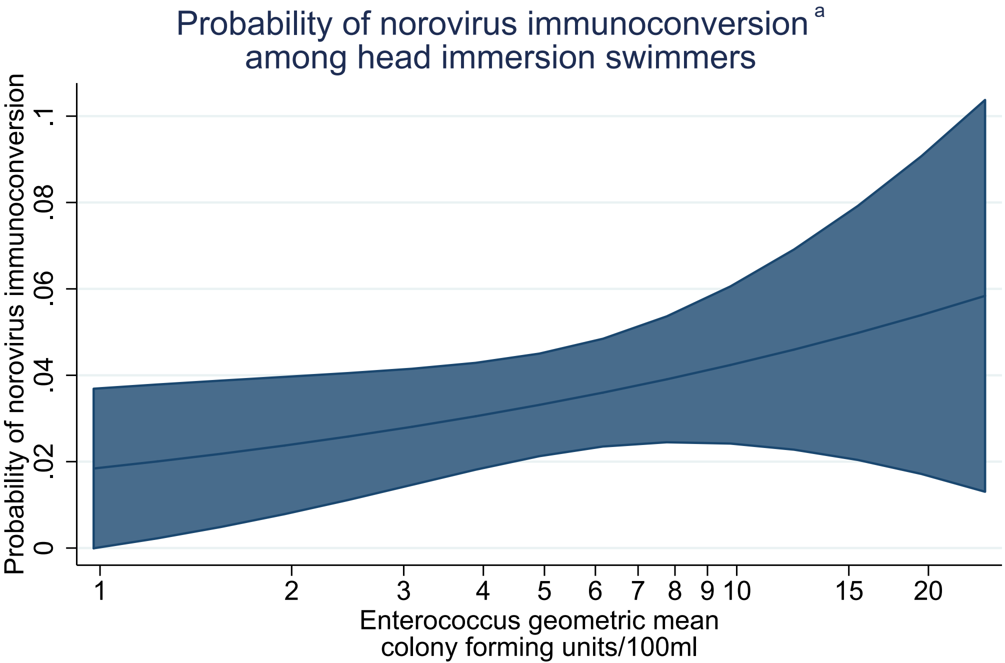

[a] Adjusted probabilities and 95% confidence limit estimated from logistic regression model
Odds ratio=2.43 (p=0.17)
