# Supplementary material for: Asymptomatic norovirus infection associated with swimming at a tropical beach: A prospective cohort study
Source: PLoS One. 2018 Mar 28;13(3):e0195056. doi: 10.1371/journal.pone.0195056 (PMC5874074; doi:10.1371/journal.pone.0195056)
Supplement: S1 Table — (DOCX) [file pone.0195056.s002.docx]

Swimming exposure and NoV immunoconversion for alternate swimming definitions

| Immunoconversion | **GI.1**  N(%) | **GII.4**  N(%) | **GI or GII.4**  N(%) |
| --- | --- | --- | --- |
| **Non-swimmers** (N=199) | 0 (0.0) | 0 (0.0) | 0 (0.0) |
| **Any contact with water** (N=1098) | 14 (1.3) | 24 (2.2) | 34 (3.1) |
| p-value^a^ | 0.15 | 0.04 | 0.006 |
| **Body immersion in water** (N=1068) | 14 (1.3) | 23 (2.2) | 33 (3.1) |
| p-value^a^ | 0.14 | 0.04 | 0.006 |

^a^ Water contact vs. non-swimmers
